# Biocidal action, characterization, and molecular docking of *Mentha piperita* (Lamiaceae) leaves extract against *Culex quinquefasciatus* (Diptera: Culicidae) larvae

**DOI:** 10.1371/journal.pone.0270219

**Published:** 2022-07-14

**Authors:** Attiya Iqbal, Naveeda Akhtar Qureshi, Saleh S. Alhewairini, Nargis Shaheen, Aneeqa Hamid, Muhammad Zahid Qureshi

**Affiliations:** 1 Department of Zoology, Entomology Laboratory, Faculty of Biological Sciences, Quaid-i-Azam University Islamabad, Islamabad, Pakistan; 2 Department of Plant Production and Protection, College of Agriculture and Veterinary Medicine, Qassim University, Buraidah, Al Qassim, Saudi Arabia; 3 Department of Pharmacy, Faculty of Biological Sciences, Quaid-i-Azam University, Islamabad, Pakistan; 4 Department of Biochemistry, Deanship of Educational Services, Qassim University, Buraidah, Al Qassim, Saudi Arabia; Universite d’Orleans, FRANCE

## Abstract

Mosquitoes are found in tropical and subtropical areas and are the carriers of a variety of diseases that are harmful to people’s health. *e*.*g*., malaria, filariasis, chikungunya, dengue fever, etc. Although several insecticides are available, however, due to insect resistance and environmental hazards, more eco-friendly chemicals are needed for insect control. So, the current research was planned to explore the prospective of *Mentha piperita* to be used for the formulation of larvicides against mosquito *Culex quinquefasciatus*. The ethanolic and water extracts of *M*. *piperita* leaves were prepared using the soxhlet apparatus. The extracts were dried and subjected to prepare five concentrations multiple of 80 ppm. Each concentration was applied for its larvicidal efficacy setting an experiment (in triplicate) in plastic containers of 1000 ml with extracts, 30 larvae of all four instars separately, and fed with dog biscuits along with controls. Observations were taken after each 12 hrs. till 72 hrs. The antioxidant perspective of *M*. *piperita* was determined by DPPH radical scavenging, total antioxidant capacity, and ferric reducing power assays. Using brine shrimp lethality bioactivity, the cytotoxic study was perceived. Standard techniques were used to classify the *M*. *piperita* extract using preliminary qualitative and quantitative phytochemicals, UV-Vis spectroscopy, FT-IR, and GC-MS analysis. *M*. *piperita* ethanolic leaves extract after 24 hrs. of exposure in 400 ppm showed 93% (LC_50_ = 208.976 ppm) mortality in ethanolic extract and 80% (LC_90_ = 246.900 ppm) in the water extract. In treated larvae, biochemical examination revealed a substantial (P<0.05) decrease in proteins, carbohydrates, and fat contents. The ethanol extract of *M*. *piperita* was the most efficient, killing brine shrimp nauplii in 50% to 90% of cases. TAC (125.4 3.5gAAE/mg DW) and FRP (378.1 1.0gAAE/mg DW) were highest in the ethanolic extract of *M*. *piperita*. The presence of medicinally active components such as alkaloids, carbohydrates, flavonoids, and others in *M*. *piperita* leaves extract in ethanol was discovered. The UV-Vis spectrum showed two peaks at 209.509 and 282.814 nm with the absorption of 2.338 and 0.796 respectively. The FT-IR consequences exhibited the occurrence of alcohols, alkanes, aldehyde, aromatic rings, ether linkage, ester, and halo- compounds. The GC-MS analysis according to peak (%) area and retention time showed ten phytochemicals consisting of six major and four minor compounds. Among all the compounds, 1, 2-benzene dicarboxylic acid, and 3-ethyl-5, 5-dimethyl -6-phenyl bound well to the NS3 protease domain with PDB ID: 2FOM. Hence, for the prevention of health hazards and mosquito control, *M*. *Piperita* is a potential source of chemicals for insecticide formulation.

## Introduction

Mosquitoes thrive in tropical and subtropical environments. Mosquitoes (Arthropoda: Insecta: Diptera) are a major public health concern because they are a vector for a variety of vertebrate diseases (pathogens) all over the world. [1]. The four most prevalent mosquito genera (*Culex*, *Anopheles*, *Mansonia*, and *Aedes*) are responsible for millions of fatalities each year due to diseases like filariasis, malaria, encephalitis, yellow fever, and dengue fever. [2]. There are 42 genera of mosquitoes, 140 subgenera, 3601 species, and subspecies worldwide, yet just 65 species belong to 06 genera (*Anopheles* (44), *Culex* (9), *Aedes* (8), *Culiseta* (2), *Armigeres* (1) and *Mansonia* (1) of mosquitoes are reported from Pakistan. WHO has stated that mosquitoes as “public enemy number one” [3]. Therefore, both vectors and vector -associated infections have developed thought-provoking difficulties that have public and economic effects [4]. Around 20% of the world’s population is at risk of getting infected with mosquito-borne diseases, which impact 700 million people each year in more than 80 countries [5].

*Culex* spp. (Diptera: Culicidae) are pan-tropical pests. It is the utmost (maximum) abundant house mosquitoes dominant in municipal and rural parts [6]. *Culex quinquefasciatus* is the most common *Culex* mosquito species in Pakistan [7], a vector of *Wuchereria bancrofti* is an urban vector, which causes filarial fever, and is primarily found in tropical areas, with 44 million people having a common chronic look and roughly 120 million persons affected globally [8].

One of the key techniques for reducing the extent of disease is to control the vector or immediate host, and this approach may be effective against juvenile or adult insect phases [9]. At the larval stages, the control of mosquitoes is vital and accomplished in cohesive mosquitos management [10].

Biological control is one of the appropriate approaches for mosquito control that is effective, simple to apply, and environmentally acceptable [11]. In the past, mosquito vectors were controlled using traditional chemical insecticides, larvicides, pupaecides, and adulticides such as carbamates, organochlorines, organophosphates, and pyrethroids. The use of traditional insecticides has negative consequences such as resurgence, insect population conflict, residual consequences, and non-specificity in action, which kills all insects, both useful and detrimental, collectively. As a result, some alternative decisions must be preferred in light of these side effects [12].

Traditional folk uses of wild plants have always prompted researchers to look into new remedies to improve human and animal health [13]. Plants are the origin of active compounds, e.g. phenolic, alkaloids, tannins, terpenoids, saponins, steroids, and flavonoids which are important to explore novel drugs [14, 15].

Natural plant products have been evaluated as prototypes for new pesticides agents because they include a rich source of bioactive chemicals that might be used in integrated management programmes [16].

Phytochemicals are detected as secondary metabolites because each fragment of the plant body may consist of active components [17]. The relationship between plant bioactivity and phytocomponents is critical to understand to develop compounds with specialized activities for treating a variety of health problems and chronic infections [18].

*Mentha piperita* is a tiny herbaceous plant with greenish feathery leaves that belongs to the family Labiatae or Lamiaceae. *Mentha* is a genus with more than 20 species. *Mentha piperita* has antibacterial, strong antioxidant, antinociceptive, anti-inflammatory, insecticidal, anticancer, and antiallergenic activity *in vitro* [19, 20].

A computer-assisted pharmaceutical technique in which two molecules are positioned in three dimensions is known as molecular docking. The molecular docking method may be used to describe the atomic level interaction between tiny molecule and protein, allowing us to define small molecular behaviour in target protein binding sites and deduce critical biochemical processes. It is the most important tool in structural biology and antilarval chemical discovery. Molecular docking methods are commonly utilized in the contemporary drug design process to understand protein-ligand interactions. Predicting the ligand structure as well as its position and orientation within these sites (known as pose) and estimating the binding affinity are the two primary processes in the docking procedure. The three-dimensional structure of the protein-ligand complex can be useful in understanding how proteins interact with one another in order to achieve biological functions. Based on its essential function in the viral life cycle, the NS3 protein is a highly promising molecular target for antiviral drugs. Since the active protease and helicase of NS3 are structurally similar, a drug that may disrupt the dynamics of this multifunctional enzyme would be appealing [21].

The recent study is focused upon the uses of *Mentha piperita* leaves extract for assessing their larvicidal properties. The current study main goal was to assess the biocidal capacity, characterization, and molecular docking of *M*. *piperita* leaves extract against *C*. *quinquefasciatus* (*C*. *fatigans*) larvae, which could be used in the future for pesticide preparation.

## Materials and methods

### Plant collection

Leaves of *M*. *piperita* L. were collected locally within the premises of Quaid-i-Azam University, Islamabad, Pakistan [22]. The herb was chosen for its pharmacological characteristics as well as its historical applications and was identified through the flora of Pakistan and taxonomists [23].

### Plant extract preparation

The foliage of plants was splashed with running water to acquire rid of debris particles and impurities. Leaves remained desiccated in the shade for 2 weeks at room temperature ranging from 27 to 37°C and were crushed mechanically with an electrical blender (Daigger Scientific USA). The 60 meshed and 40 retained materials of leaves were selected for extraction. In 300 ml of two different solvents, 30 g powdered material of each plant was eluted i.e. ethanol and water in a Soxhlet extraction device (Shanghai Heqi^®^, China) for 6 to 8 hrs (two cycles each hr). The filtrates were sieved with the use of Whatman filter paper No. 1. The dried filtrates were collected by vaporizing the solvent with a rotary vacuum evaporator (R-300, Rotavapor^®^, Germany) and stored at 4° C in airtight bottles to make stock solutions.

One gram of crude extract was dissolved in 100 ml of distilled water to make a stock solution. Various concentrations (80, 160, 240, 320, and 400 ppm) were produced from the stock solution using the formula [24].


$${\text{C}}_{1}{\text{V}}_{1}={\text{C}}_{2}{\text{V}}_{2}$$

C_1_ = Stock solution conc. (ppm) V_1_ = Required volume

C_2_ = Required conc. V_2_ = Given volume

### Larvae collection and bioassay

*Culex quinquefasciatus* larvae were obtained using the dipping method from natural ponds around Quaid-i-Azam University in Islamabad, Pakistan, during the pre-monsoon season [25]. Larvae were identified using the key [26] and preserved in plastic containers containing tap water with net casing cultures in dark and light settings at 262° C and 70–80% RH. The I^st^, 2^nd^, 3^rd^, and 4^th^ instars larvae were detached and saved in a 1000 ml plastic container. Thirty larvae were released using a dropper in each container holding 200 ml of plant extracts at varying concentrations (80, 160, 240, 320, and 400 ppm). With each concentration, a control (distilled water only) was likewise set up. Ethanol was also used as a positive control. Each container was covered with mosquito netting and the larvae were fed with crushed dog biscuits and yeast (3:1). For each concentration sideways, the experiment was repeated three times with no extract as a control. Every 24 hrs until 72 hrs, the average larval mortality and observations were recorded. The morphological alterations in the experimental and control larvae were observed with a Trinocular microscope (Optika^®^ 500, Italy).

### Biochemical analysis of experimental larvae

Dead larvae from each concentration were taken after 48 hrs of exposure, eroded with saline solution, dried, and weighed using an electric scale. Sucrose solution (0.25M) was used to homogenize 90 mg of each sample using a homogenizer. The homogenate was centrifuged at 13000 rpm for 15–20 mins. The supernatant was collected and stored at -20° C. For the estimation of proteins (lowrys’ method) employing bovine serum albumin (BSA) as the standard [27], carbohydrates (phenol sulphuric method) [28] sucrose taken as standard, and lipids (cholesterol, triglycerides, and high-density test) (biochemistry analyzer) (Motenu^®^, China).

### Cytotoxic bioassay

Shrimp eggs were placed on one side of the partitioned tiny tank, which was then filled with artificial salt water (38 g NaCl/1000 ml tap water). Before being used for bioactivity and nauplii growth, the shrimps were allowed to hatch for 48 hrs. The ethanolic extract of *M*. *piperita* was taken at different quantities (5, 7.5, 10, 12.5, and 15 mg/10 ml) in sample tubes. Ethanolic extract was dissolved in DMSO for the cytotoxic assay. A disinfected pipette was used to deliver 20 live shrimps into each test tube, along with the control group. After 24 hrs, the tubes were examined with a magnifying glass for counting of alive nauplii in each vial, and interpretations for each container were recorded. Each test consisted of five recurrences that were repeated three times. We calculated the LC_50_, 95% confidence limit, LC_90_, and chi-square values. As a positive control, conventional tricaine methanesulfonate was used [29].

### Antioxidant assay

DPPH (2,2-diphenyl-1-picrylhydrazyl) radical-scavenging, total antioxidant, and ferric reducing assay were measured according to the standard protocols [30].

### Characterization

#### Preliminary phytochemical qualitative and quantitative screening

According to conventional methods, a preliminary phytochemical qualitative screening for several secondary metabolites was performed on the ethanolic extract of *M*. *piperita* (S1 Table) [31–38]. The color intensity or precipitate formation was used as analytical response to these tests.

Standard procedures were used to conduct a quantitative phytochemical study of *M*. *piperita* (S2 Table) [39–41]. A spectrophotometer was used to determine the quantity of these phytoconstituents.

#### Ultraviolet-visible spectroscopy (UV-Vis)

Under UV and visible light, the extract was scanned for proximate evaluation and centrifuged at 300 rpm for 10 mins before sieving through Whatman No.1 filter paper. Using a similar solvent, dilute the sample to 1:10 The characteristic peaks, which ranged in wavelength from 200 to 800 nm, were detected using a spectrophotometer (Perkin Elmer, USA Model: Lambda 950) [42].

#### Fourier-transform infrared spectroscopy (FT-IR)

For the detection of characteristic peaks and their functional groups, FT-IR analysis of the plant extract was obtained using Shimadzu FT-IR– 8400s Germany Vertex 70 infrared spectrometer. For the preparation of the transparent sample disc in 100 mg of KBr pellet, the extract powder in dry form (10 mg) was crushed. The IR scan was carried out in the wavenumber region of 400–4000 cm^-1^. The peak values of FT-IR were recorded [43].

#### Gas chromatography-mass spectrometry (GC-MS)

The GC-MS analysis was performed using a Shimadzu GC-201 with auto-injector AOC-20i, autosampler AOC-20s, and a gas chromatograph equipped with a QP2010ultra mass-selective detector. A fused-silica capillary column DB-5MS (0.25 mm, 30 mm, 0.25 mm) was employed for screening, with a maximum temperature capacity of 325°C. The structure was confirmed using electron impact mass spectra (EI spectra). 70 eV EI ionization was utilized with a 1.2 kv electron multiplier setup in full scan operation mode (m/z = 50 to 350) for peak detection and quantification.

The mass-selective detector was auto-tuned using perfluorotributylamine (PFTBA) with mass m/z 69, 219, and 502. After the injection, the chromatograph was set to split injection system mode with a purge flow of 3.0 ml/min. The injection volume was 1.0 l, with a split ratio of 10:1. At a flow rate of 1.0 ml/min, highly pure helium gas was used as a carrier gas. The injector was heated to 250°C, while the interface was heated to 290°C. The temperature of the column was initially fixed at 100°C. The temperature was maintained at 100°C for 0.5 mins after injection, then increased at a rate of 24°C/min to 280°C for 3 mins, for a total run time of 11 mins [44].

#### Phytoconstituents identification

The mass spectrum was analyzed using the National Institute of Standards and Technology (NIST) database, which has approximately 62000 patterns. The NIST library’s relationship between spectra of recognized and unidentified compounds aids in understanding the name, chemical formula, chemical structure, molecular weight, and mass spectrum of various substances [45].

### Molecular docking

#### Building the receptor

The 3D structure of the receptor was replicated and altered from the protein data bank (PDB) [46]. After alteration of the receptor, the accessible characteristics involved, such as stabilizing charges, elimination of water molecules from the cavity, formation of side chains and filling in empty residues, etc., are biologically active and stable. The structure of the NS3 protease domain, PDB ID: 2FOM, was obtained from the protein data bank.

#### Identification of the active site

The active site was recognized after building the receptor. One of the active sites was designated because the receptor has many sites. Many water molecules and hetero- atoms were removed. The MOE (molecular operating environment) is a computer-generated drug design screening software that is used to provide lead compounds and analyze the binding affinity of test compounds with protein NS3 in the form of E- values versus potential drug targets. To give the maximum surface area for chemical binding, a three-dimensional grid with coordinates (x, y, and z) was designed, as well as extra default parameters for MOE docking [47].

#### Ligand preparation

Ligands can be found in databases like ZINC, and Pubchem can be drawn using programs like Chemsketch [48].

#### Docking

The ligand has been docked onto the receptor, and the interactions have been verified. The scoring function assigns scores based on the ligand with the best fit that was chosen. Docked compounds screened against 1, 2-Benzenedicarboxylic acid and 3-ethyl-5, 5-dimethyl -6-phenyl of *Culex quinquefasciatus* [49].

#### Statistical analysis

To determine whether there was a significant difference between plant extracts, larval stages, concentrations, and time length, the mean mortality data of larvae was subjected to one-way analysis of variance (ANOVA) with Tukey’s multiple comparison tests using "R language. Probit analysis [50] was also used to calculate LC_50_, LC_90_, 95% confidence limits (upper and lower), and chi-square using Minitab 18 software. Statistical significance was determined when the p-value was less than 0.05.

## Results

### Effect of hours, concentrations, and plant on average mortality of its I^st^, 2^nd^, 3^rd^, and 4^th^ instars larvae in ethanol and water solvent

**Fig 1:**
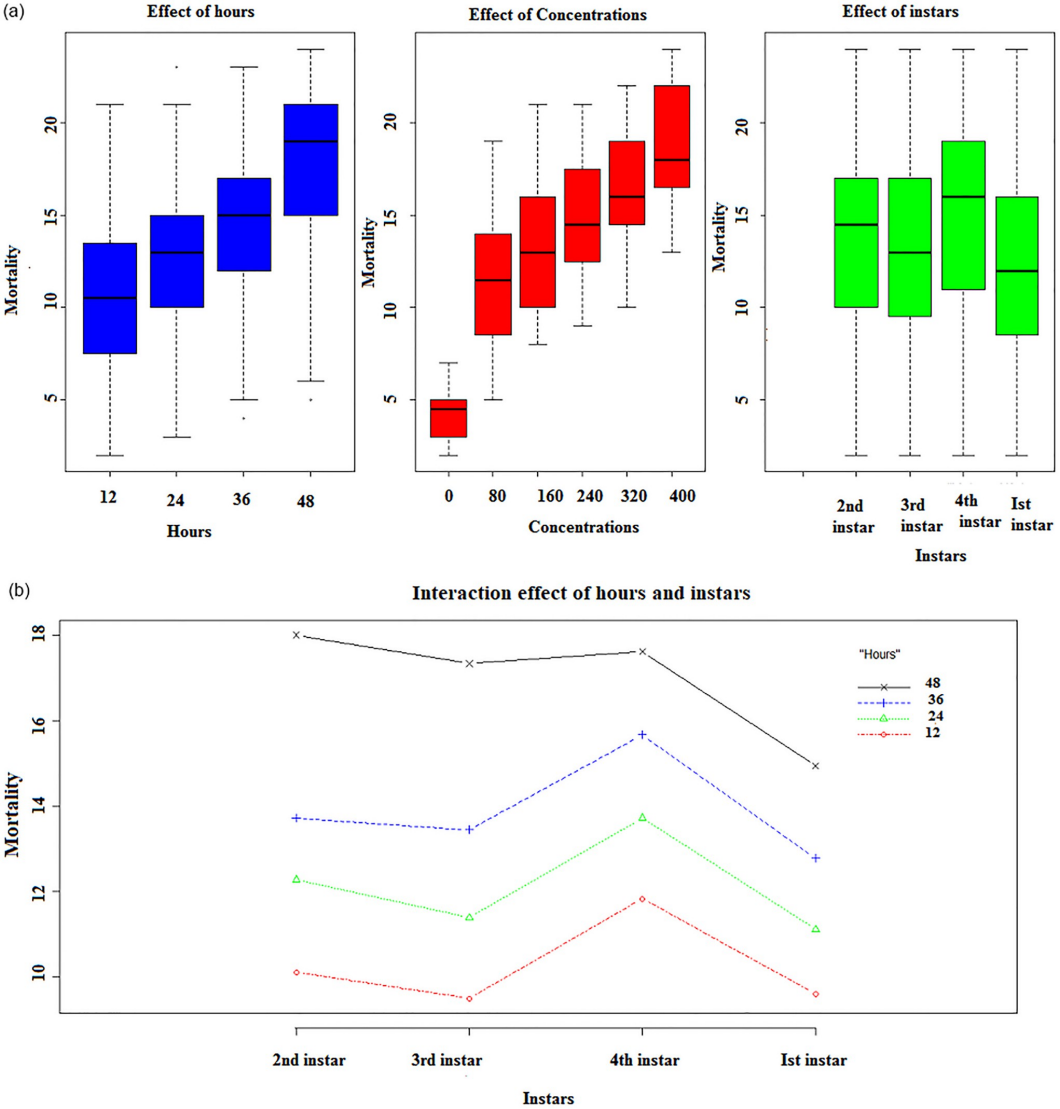
(a) Box plot representing the effect of hours, concentrations, and plant on the mean mortality of I^st^, 2^nd^, 3^rd^, and 4^th^ instars larvae in ethanol solvent. (b) Interaction plot representing the interacting effect of hours with instars on the mean mortality of I^st^, 2^nd^, 3^rd^, and 4^th^ instars larvae in ethanol solvent.

**Fig 2:**
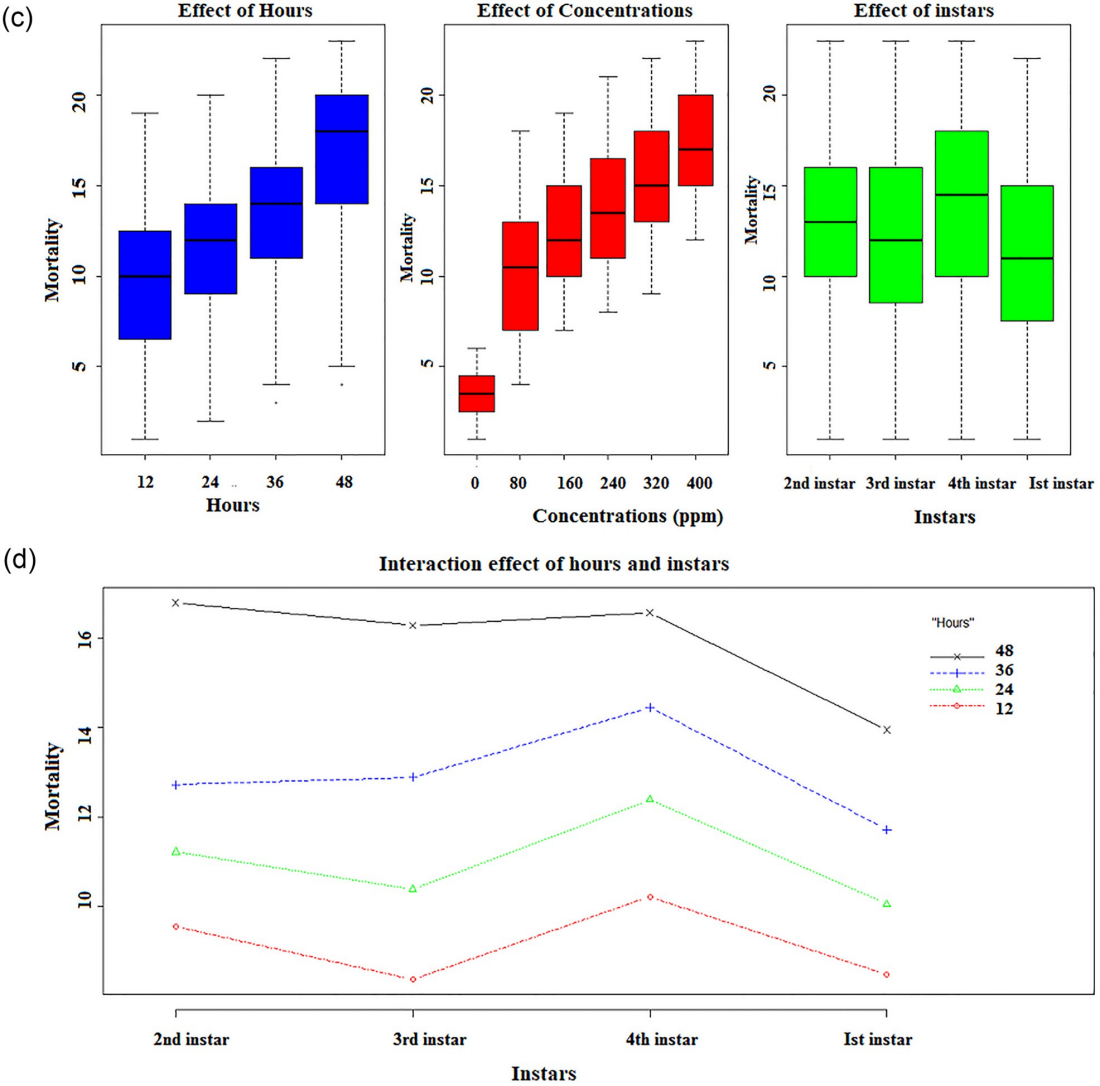
(c) Box plot representing the effect of hours, concentrations, and plant on the mean mortality of I^st^, 2^nd^, 3^rd^, and 4^th^ instars larvae in a water solvent. (d) Interaction plot representing the interacting effect of hours with instars on the mean mortality of I^st^, 2^nd^, 3^rd^, and 4^th^ instars larvae in a water solvent.

The ethanolic extract of *M*. *Piperita* revealed prominent larvicidal activity at different levels against all instars larvae of *C*. *quinquefasciatus*. The mean larval mortality at 12, 24, 36, and 48 hrs after exposure to the five different concentrations of each ethanolic and aqueous plant extract (Figs 1 and 2).

In a time-dependent manner, all ethanolic doses were observed to have a significant influence on larval mortality when compared to control (F (DF) = 445.11(5); P< 0.05) that differed considerably from one another. Fig 1 depicts the influence of days, concentrations, and plants on the mean mortality of larvae. As the concentration of the ethanolic and aqueous plant extract of *M*. *piperita* grew, so did the number of deaths happen, and will also affect the change of instars (Ist instar>2^nd^ instar>3^rd^ instar>4^th^ instar). The same concentration related to different instars affect the metabolic rate differently. S3 Table shows the results of a three-way factorial ANOVA of larval instars conducted at various doses and time intervals that revealed significant variations in larval mortality (P< 0.05).

The Chi-square test, Tukey’s test, and the 95% confidence level were used to compare mean percentage mortality with standard error and upper and lower bounds, and the values were significant at P< 0.05 (Table 1). The P-value of I^st^, 2^nd^, 3^rd^ and 4^th^ instar larvae in ethanol (0.001, 0.00, 0.003, 0.001) and water extract (0.01, 0.04, 0.05, 0.004). Among them, an ethanolic extract exhibited the highest larvicidal activity. The water extracts also produced similar results with non -significant differences from ethanolic extracts of the same plant.

**Table 1:** Probit analysis (LC_50,_ LC_90_), Chi- square and P-value of experimented plant.

Plants Name	Larval instars	LC_50_ (UCL-LCL) (PPM)		LC_90_ (UCL-LCL) (PPM)		P- value		Chi–square (χ2-value)	
		Ethanol	Water	Ethanol	Water	Ethanol	Water	Ethanol	Water
*Mentha piperita*	Ist instar	290.688 (314.631–270.030)	300.612 (353.395–302.582)	330.440 (318.309–363.456)	368.512 (364.617–396.161)	0.001	0.01	10.72	15.45
	2^nd^ instar	151.434 (274.974–230.173)	234.767 (311.256–262.141)	346.149 (349.071–370.431)	379.371 (388.741–399.179)	0.00	0.04	46.30	52.20
	3^rd^ instar	213.226 (298.971–250.892)	275.755 (334.842–281.652)	304.245 (383.985–393.965)	323.888 (319.072–319.794)	0.003	0.05	35.64	41.24
	4^th^ instar	208.976 (225.926–192.225)	246.900 (265.704–229.445)	319.576 (359.605–352.395)	349.374 (318.088–395.929)	0.001	0.004	36.45	41.03

LC = Lethal concentration, UCL = Upper confident limit, LCL = Lower confident limit, PPM (parts per million)

### Biochemical test

**Fig 3:**
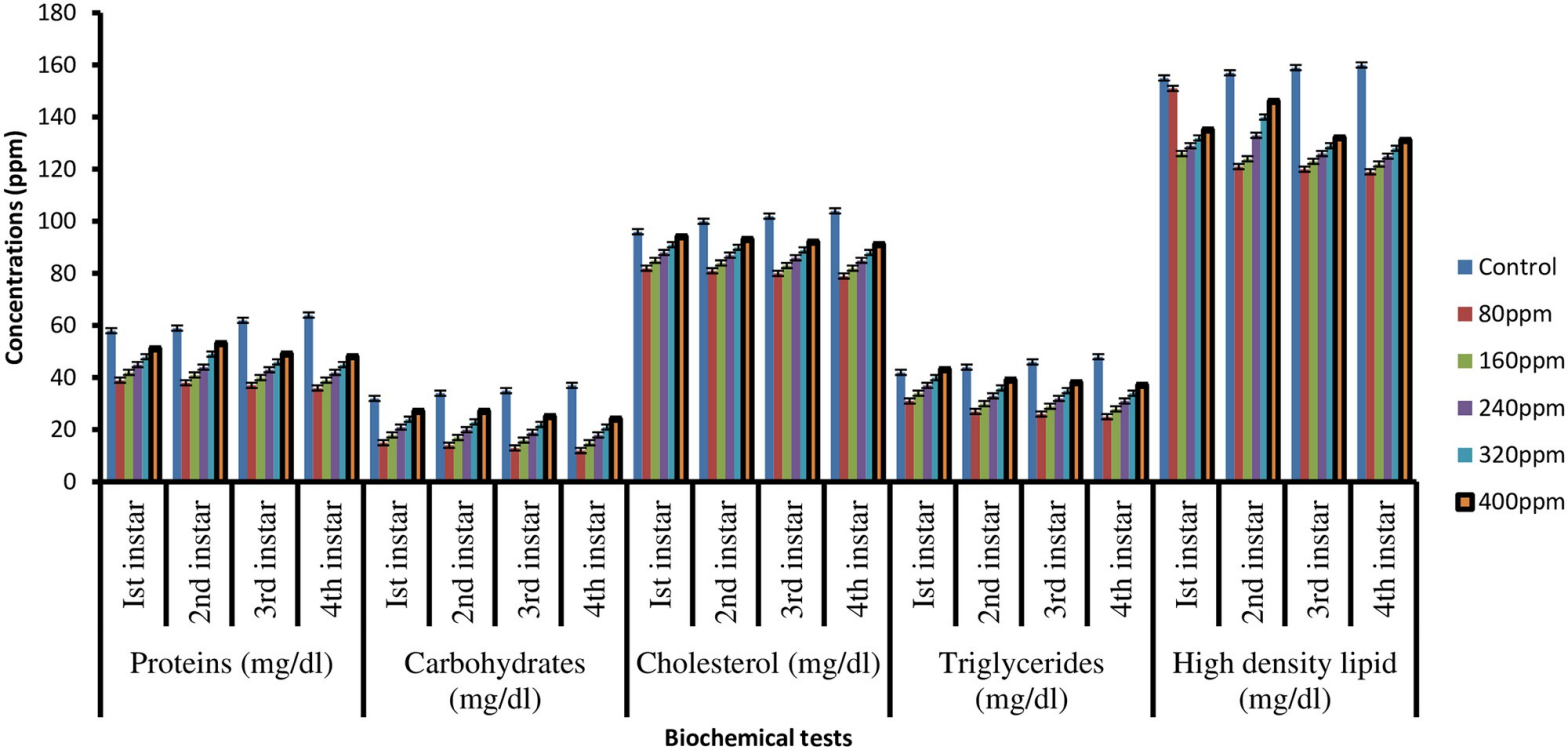
Proteins, carbohydrates, cholesterol, triglycerides and high density lipid estimation of I^st^, 2^nd^, 3^rd^, and 4^th^ instars larvae of *C*. *quinquefasciatus* in different concentrations of *M*. *piperita* leaves extract in ethanol solvent.

**Fig 4:**
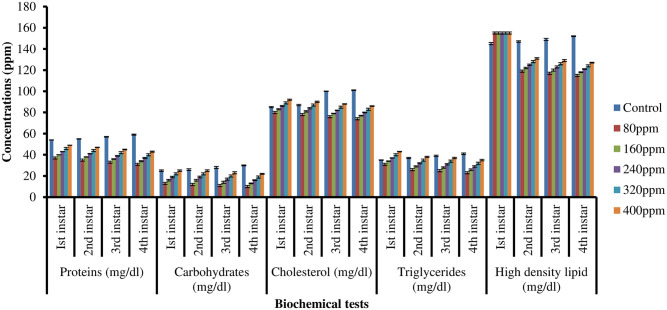
Proteins, carbohydrates, cholesterol, triglycerides and high density lipid estimation of I^st^, 2^nd^, 3^rd^, and 4^th^ instars larvae of *C*. *quinquefasciatus* in different concentrations of *M*. *piperita* leaves extract in a water solvent.

Biochemical analysis of all instars larvae treated with *M*. *piperita* extract in ethanol and water solvent revealed considerably lower levels of proteins, carbohydrates, and lipids (triglyceride, cholesterol, and high-density lipid) than in control (Figs 3 and 4).

### Brine shrimp cytotoxicity assay

The ethanolic extracts of *M*. *piperita* showed that with the increasing concentrations of the extract, the brine shrimp mortality rate also increases. The deadly chemicals in the crude extracts were shown to have an inhibiting effect. The ethanolic extract was determined to be the most effective, resulting in 50% brine shrimp nauplii mortality (Table 2). The cytotoxicity of the water extract was lower. Brine shrimp nauplii were found to be completely dead in the standard.

**Table 2:** Concentration dependent cytotoxic potential of crude ethanolic and aqueous extracts of *M*. *piperita* leaves against Brine shrimps nauplii.

Plant extract	Concentrations (ppm)	Number of shrimps	Number of shrimps killed	Mortality (%)	LC_50_ (ppm)
Ethanol	80	20	5	25	22.24±0.45
	160	20	8	40	20.21±0.34
	240	20	14	70	17.37±0.25
	320	20	16	80	15.45±0.11
	400	20	18	90	8.74±0.04
Water	80	20	3	15	37.11±0.55
	160	20	6	30	32.56±0.36
	240	20	10	50	27.12±0.27
	320	20	12	60	25.45±0.13
	400	20	15	75	15.87±0.10

### Antioxidant assay

The TAC (total antioxidant capacity) of the ethanolic extract was determined and represented as AAE g/mg DW (ascorbic acid equivalent). The TAC of ethanolic extracts of *M*. *piperita* (125.4 ± 3.5 μgAAE/mg DW) is higher than water extract (275.3±2.2). These consequences showed that high antioxidant potential was present in *M*. *piperita* ethanolic extract as compared to aqueous extract.

The ethanolic plant extract from *M*. *piperita* indicated the maximum FRP (ferric reducing power) values (378.1 ± 1.0 μgAAE/mg DW) as compared to water extract (275.3 ± 2.2 μgAAE/mg DW). According to a prior study, a plant extract’s reducing power capacity is proportional to its phenolic concentration. To test its antioxidant activity, researchers compared the ability of an ethanolic extract of *M*. *piperita* to scavenge DPPH free radicals to vitamin C. At increasing extract concentrations, ethanol’s DPPH free radical scavenging activity was higher than that of water extract. But at 400 ppm, *M*. *piperita* showed maximum antioxidant potential (85.43 ± 1.22) through scavenging which exhibited that *M*. *piperita* may comprise some compounds that have a higher dose effect. The IC_50_ of *M*. *piperita* ethanolic extract is 15.57 which is lower than compared water extract (Table 3).

**Table 3:** DPPH free radical scavenging (%) activity from *M*. *piperita* of ethanolic crude extracts.

Concentrations (μg/ml) % inhibition by *M*.*piperita* and Vitamin C											IC_50_ μg/ml	
Plant name	80ppm		160ppm		240ppm		320ppm		400ppm			
	Ethanol	water	Ethanol	water	ethanol	Water	Ethanol	Water	Ethanol	Water	Ethanol	Water
*M*. *piperita*	63.84±1.34	53.48±1.44	65.75±1.14	55.57±1.24	68.63±1.42	67.26±1.54	75.74±1.75	73.48±1.77	85.43±1.22	78.33±1.42	15.57	20.58
Vitamin C	66.23±1.05	56.23±1.25	69.26±1.21	58.62±1.29	73.65±1.00	71.54±1.11	87.54±1.28	79.23±1.30	103±1.09	85.12±1.19	6.51	8.52

DPPH = 2,2-diphenyl-1-picrylhydrazyl, IC = inhibitory concentration, Microgram per milliliter (μg/ml)

### Characterization

#### Preliminary qualitative and quantitative phytochemical analysis

Several bioactive secondary components were discovered in the qualitative phytochemical investigation of *M*. *piperita*, which could be responsible for their therapeutic properties. Major components were found in the leaves extracts of *M*. *piperita* in ethanol are alkaloids, flavonoids, phenols, etc. A phytochemical qualitative study of the leaves extracts of *M*. *piperita* in water revealed the presence of pharmaceutically active elements such as tannins, phenols, alkaloids, etc (Table 4).

**Table 4:** Qualitative and quantitative phytochemical analysis of *M*. *piperita* in ethanolic and water extracts.

Phytochemical Compounds	Ethanol	Water	Mean ± STD (Ethanol)	Mean ± STD (water)
Alkaloids	++	++	5.43±0.0023	3.33±0.0023
Carbohydrates	+	+	2.68±0.0028	1.58±0.0028
Flavonoids	++	+	3.47±0.0017	1.37±0.0017
Cardiac glycosides	+	+	3.55±0.0015	1.45±0.0015
Saponins	+	-	3.67±0.0030	2.47±0.0030
Tannins	+	++	4.70±0.0031	2.50±0.0031
Reducing Sugar	+	+	4.21±0.0027	2.11±0.0027
Sterol	-	-	-	-
Quinones	+	+	-	-
Terpenoids	+	-	1.97±0.0029	0.87±0.0029
Phenol	++	++	6.44±0.0018	4.24±0.0018
Amino Acid	+	-	-	-
Volatile Oil	+	-	-	-
Starch	+	+	-	-
Cellulose	+	+	-	-
Anthocyanin	+	-	-	-
Betacyanin	+	-	-	-
Anthraquinones	+	-	-	-
Coumarins	-	-	-	-
Phlobatannins	+	-	-	-
Steroids	+	+	0.66±0.0021	0.36±0.0021
proteins	+	+	2.37±0.0025	1.27±0.0025
Phytosteroid	+	-	-	-
Leucoanthocyanins	-	-	-	-

STD = (Standard deviation), + = Present,— = (Absent), ++ (Highly present)

The quantitative phytochemical analysis of an ethanolic extract of *M*. *piperita* showed the degree of abundance of these phytochemicals in percentage are as follows; 6.44 ± 0.0018 phenols, 5.43 ± 0.0023 of alkaloids, and 4.70 ± 0.0031 of tannins etc The quantitative phytoconstituents in the water extract of *M*. *piperita* are as follows; 4.24 ± 0.0018 of phenols, 3.33 ± 0.0023 of alkaloids, 2.50 ± 0.0031 of tannins, etc (Table 4). The phytochemical with the maximum quantity was phenol, followed by alkaloids, tannins, reducing sugar, saponins, cardiac glycosides, flavonoids, carbohydrates, proteins, terpenoids, and steroids respectively.

#### UV-Vis spectroscopy

**Fig 5:**
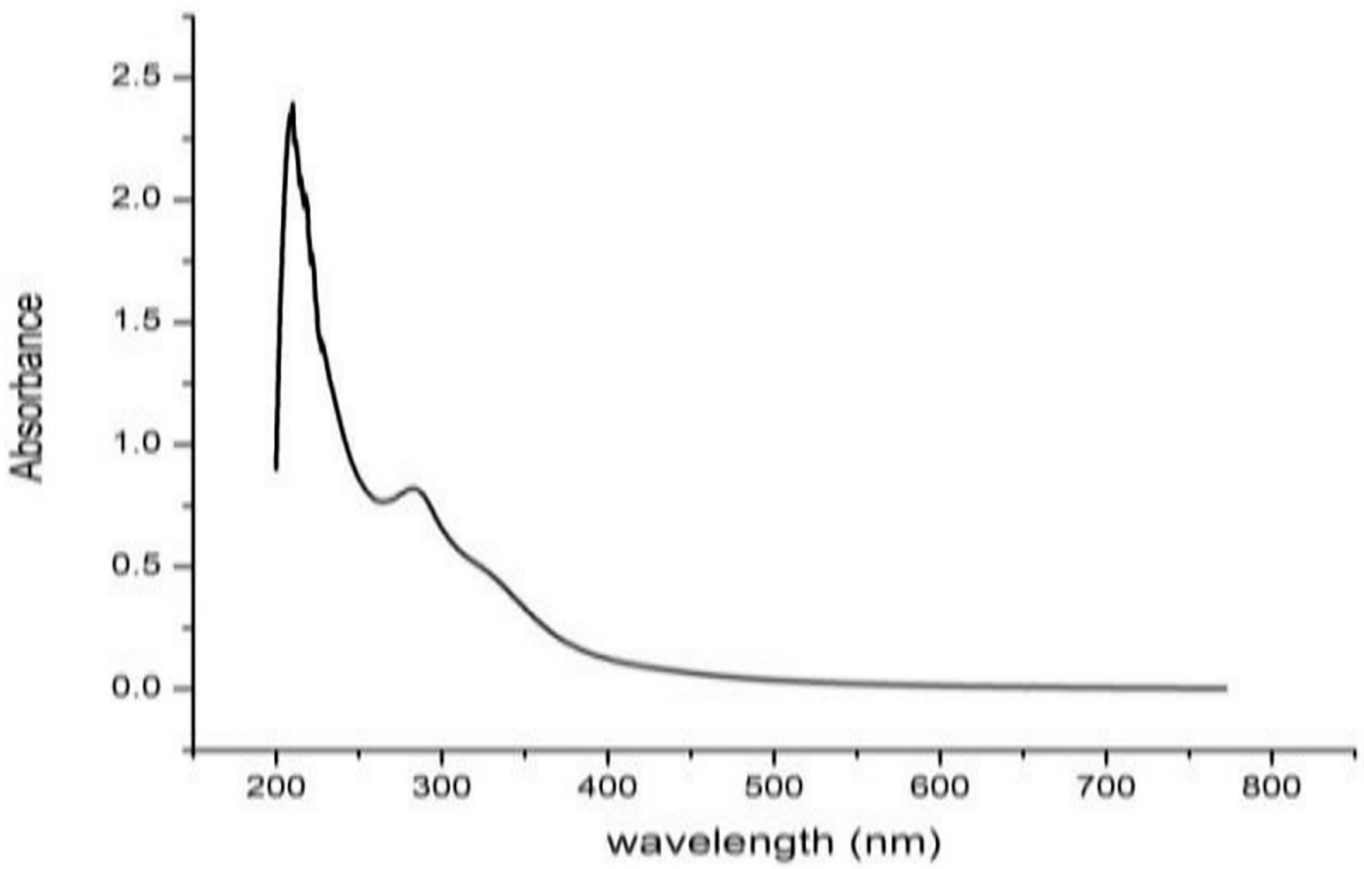
UV-Vis spectrum of ethanolic extract of *M*. *piperita*.

For identification of phytoconstituents present in crude extract, the UV-Vis study was accomplished. Due to the sharpness of the peaks and proper baseline at the wavelength of 200 to 800 nm, a spectrum profile was selected. The UV-Vis spectrum exhibited two peaks at 209.509 and 282.814 nm with the absorption of 2.338 and 0.796, respectively. This confirms the presence of unsaturated groups and heteroatoms such as N, S, and O (S4 Table and Fig 5).

#### FT-IR spectroscopy

**Fig 6:**
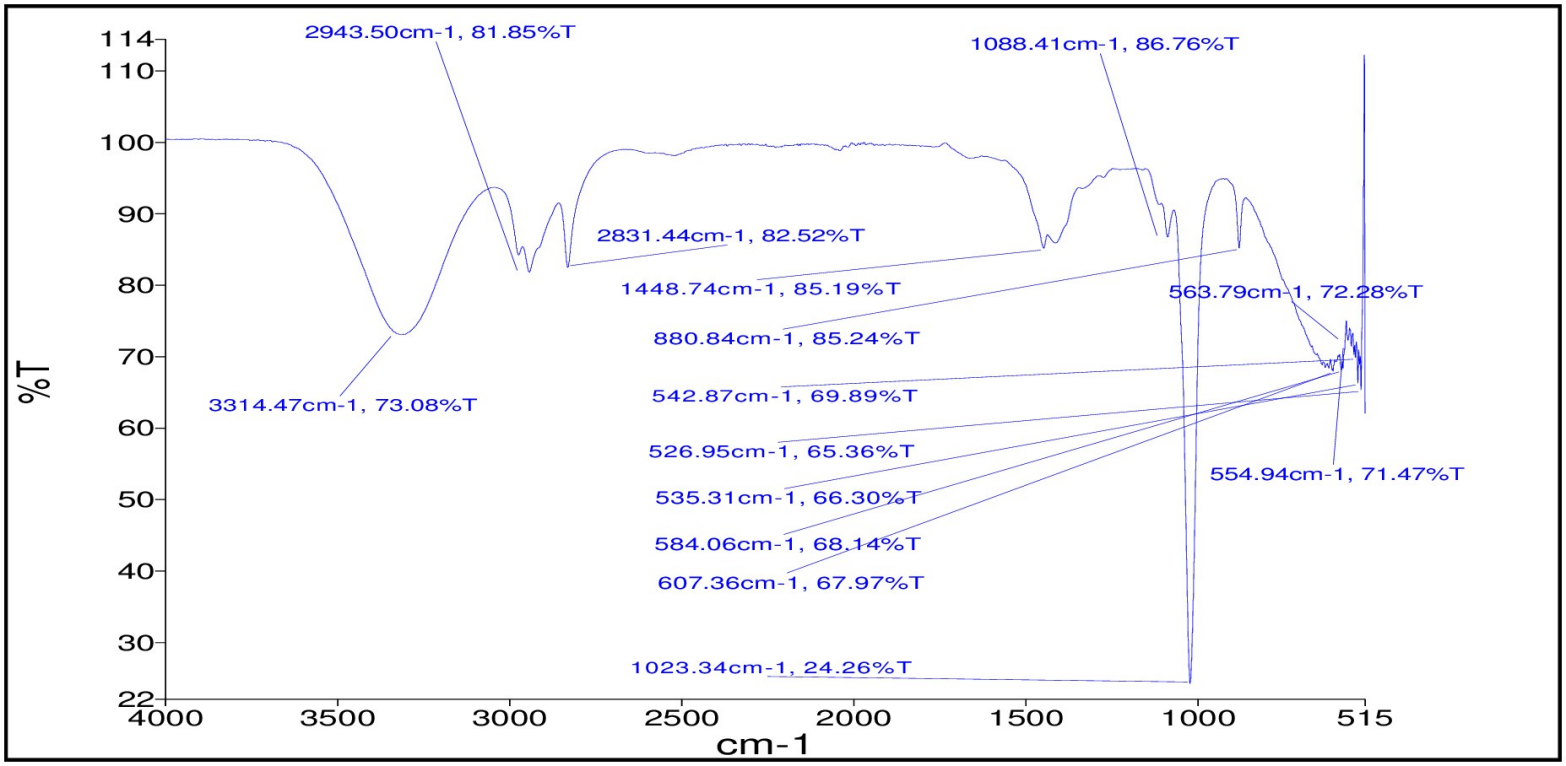
FT-IR spectra of ethanolic extract of *M*. *piperita*.

For the detection of functional groups of the active constituents in the area of infrared radiation, the FT-IR spectrum was used. The consequences of FT-IR investigation confirmed the presence of alcohols, alkanes, aldehyde, aromatic ring, ether linkage, ester, and halo- compounds. FT-IR spectra revealed the presence of functional groups:—O-H, C-H, C = = C, C-F, C-O-C, C-Br, and C-I (Fig 6).

#### GC-MS

Gas chromatography coupled mass spectroscopy of ethanolic leaves extracts of *M*. *piperita* specified ten peaks which showed the presence of ten phytochemicals. GC-MS is the convenient method to detect the components of volatile matter, long and branched-chain hydrocarbons, ester, ketones, carboxylic acid, alcoholic acid, aldehyde, terpenes, substituted alkenes, phenolic compounds, heterocyclic nonaromatic compound, plasticizer, and monoterpenes ketone. Retention time, peak area %, molecular formula, molecular weight, compound nature, uses, and biological activity for the identification of phytochemical components. T-Butyl hydrogen phthalate (13.99%), 2H-Pyran-2,4(3H)-dione,3-ethyl-5,5-dimethyl -6-phenyl (13.15%), 1,2-Benzenedicarboxylic acid, mono-(2-Ethylhexyl) ester (12.45%), Olean-12-en-28-oic acid, 2.beta., 3.beta.,23-trihydroxy-methyl ester (10.74%), 1,2-Benzene dicarboxylic acid, butyl octyl ester (10.36%), 8-Octadecen-1-ol acetate (9.64%) are reported as a major constituents in ethanolic leaves extract of *M*. *piperita* while 2-Butanone, 4-(2,6,6-trimethyl-1-cyclohexen-1-yl)- (7.75%), Pentadecanal (7.48%), 2-Cyclohexen-1-one,5-methyl-2-(1-methylethyl) (7.45%), 2-Napthalenamine, 1,2,4a,5,6,7,8,8a-octahydro-4a-methyl (7.29%) are present as minor compounds in *M*. *piperita* leaves extract. These phytochemicals are responsible for various pharmacological actions like antimicrobial, antibacterial, antifungal, mutagenic, phytotoxic, anti-oxidant, anticancer activities, etc (S5 Table and S1 Fig).

#### Molecular docking

NS3 protease’s three-dimensional crystal structure has been designated for docking. At the N-terminal domain, the zinc-dependent serine protease NS3 protease activity is localized. The zinc ion, which is required for hydrolytic activity, is thought to be a structural metal ion that is necessary for the protein’s structural integrity. NS3 also interacts with another cofactor, NS4A, a supplementary viral protein that causes a conformational shift that improves the hydrolytic activity. At the ligand, the grid center was fixed, and grid energy measurements were done.

To justify the antilarval potency of the ten compounds from *M*. *piperita*, their binding affinity with NS3 was assessed. The best docked ligand-protein complex, as defined by best binding affinity values, hydrogen bond number, and hydrogen bonding residues. Out of all the compounds. 1, 2-Benzenedicarboxylic acid and 3-ethyl-5, 5-dimethyl -6-phenyl had the greatest E values (-6.9107 and -6.8340) kcal/mol, respectively.

The hydrogen bonds of compounds 1,2-Benzenedicarboxylic acid and 3-ethyl-5,5-dimethyl-6-phenyl with NS3 protease are shown in 2D and 3D interaction diagrams. For 1,2-Benzenedicarboxylic acid and 3-ethyl-5,5-dimethyl-6-phenyl, the key contact residues were (Leu, Ala, Gly, Val, lle, trp, Asn, lys, Thr) and (Lys, Val, Thr, lle, Asp, Asn, Ala, Met, Trp, Leu, Gly,) respectively.

Both compounds bind to the NS3 protease and interact with it through a variety of chemical forces. One hydrogen bond between the compound’s acidic hydrogen and the protein’s electronegative component, two between the electronegative oxygen and the positive element of the residue protein, and a fourth between the compound’s electronegative nitrogen and the residual protein appear to be complex.

Many other significant electrochemical forces, such as Van der Waals, carbon-hydrogen interaction, covalent link, and so on, are also involved. The process of covalent inhibition is irreversible. The irreversible inhibitors work in concert with their specific targets in a time-dependent way, and the reaction eventually comes to close before reaching equilibrium.

**Fig 7:**
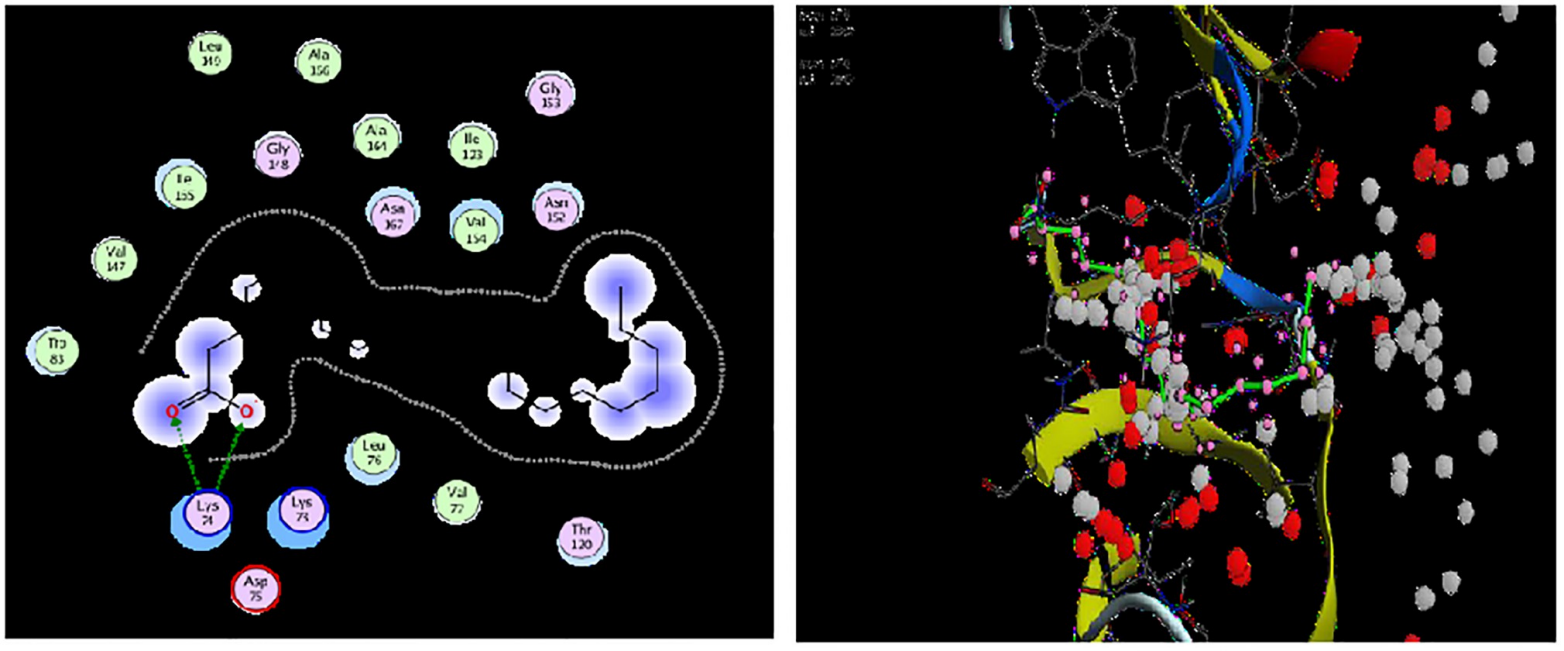
2D and 3D interaction of compound (1, 2-Benzenedicarboxylic acid) with the target protein (PDB ID: 2FOM).

**Fig 8:**
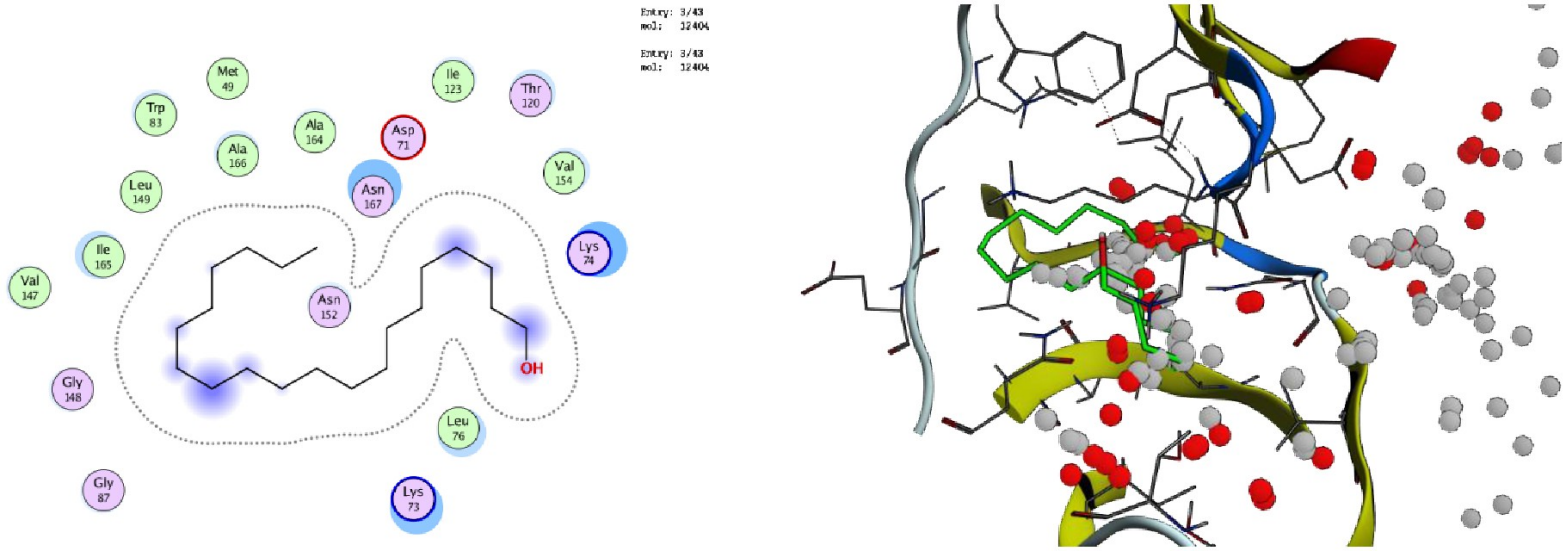
2D and 3D interaction of compound (3-ethyl-5,5-dimethyl -6-phenyl) with the target protein (PDB ID: 2FOM).

This research suggests that the 1, 2-Benzenedicarboxylic acid, 3-ethyl-5,5-dimethyl -6-phenyl, and the protein can have a strong association based on electrochemical contact forces. The compounds 1, 2-Benzenedicarboxylic acid, and 3-ethyl-5,5-dimethyl-6-phenyl were found to bind to the NS3 receptor effectively. Only two compounds, 1-Benzenedicarboxylic acid and 3-ethyl-5, 5-dimethyl-6-phenyl, showed meaningful results out of ten (Figs 7 and 8).

## Discussion

Vector control activities are currently focused on mosquito larval elimination using natural materials (plant derivatives) as an alternative to synthetic insecticides. Mosquito larvae have the advantage of not being able to leave their target/breeding areas until they reach adulthood. Larvicides are favored over insecticides because they are more selective and have a lower LC_50_ [51].

There is a great desire to use natural, environmentally acceptable chemicals for larvicidal activity on a global scale. In many nations, the mortality of millions of people each year as a result of mosquito-borne diseases has resulted in a loss of socioeconomic prosperity. At this time, chemical mosquito control is considered to be dangerous. Insecticides are incompatible with vectors and cause environmental imbalances. Chemical or synthetic pesticides have long-term harmful impacts and consequently do not deliver 100% effective results [52].

Consequently, alternative mosquito control approaches are necessary. Poisonous and other dynamic components are frequently recognised as part of the therapeutic paradigm of crude plant extracts. For identifying the potential and active components in crude plant extract against mosquito larvae, primary screening is a good concept. When compared to other experimental plants, the ethanolic extract of *M*. *piperita* was substantially (P<0.05) active in causing the highest average mortality in each larval stage of *C*. *quinquefasciatus* [53].

Although the water extract had substantial (P<0.05) anti-mosquito larval activity, it was less than the ethanolic extracts. The increased solubility of mint in ethanol, as well as the presence of flavonoids and anthraquinones, may account for its larvicidal affect. Flavonoids extracted from the crude extracts of *Vitex negundo* and *Andrographis paniculata* were found to cause considerable mortality in *Aedes aegypti* and *Anopheles stephensi* larval instars III and IV [54].

Flavonoids and phenols identified in aqueous crude extracts of *Hemidesmus indicus*, *Gymnema sylvestre*, and *Eclipta prostrata* totally killed *C*. *quinquefasciatus* mosquito larvae at a concentration of 5% [55]. A study [56] looked into the efficiency of 31 plant extracts from Europe and Asia in killing *C*. *quinquefasciatus* larvae. Six plants were well-known as effective larvicides due to the presence of flavonoids in another investigation, which examined petroleum ether extracts of sixty-three plants for larvicidal efficiency against larvae of *C*. *quinquefasciatus*, *Anopheles stephensi*, and *Aedes aegypti* [57].

Based on the average percent mortality of *C*. *quinquefasciatus* larvae, to estimate the toxicity of each plant extract, the LC_50_ and LC_90_ were determined using Probit analysis. The LC_50_ of *M*. *piperita* water extract was 301.90, 276.60, and 198.02 ppm for 2^nd^, 3^rd^, and 4^th^ instar larvae, respectively, and 312.22, 390.92, and 313.75 ppm for *O*. *basilicum*. For 2^nd^, 3^rd^, and 4^th^ instar larvae, *M*. *piperita* and *O*. *basilicum* water extract had LC_90_ of (329.67, 303.43, 337.81 ppm) and (329.21, 377.52, and 374.22) for 2^nd^, 3^rd^, and 4^th^ instar larvae, respectively [58].

When compared to control (F (DF) = 445.11(5); P 0.05) that differed significantly from one another, all ethanolic doses were seen to have a substantial effect on larvae mortality in a time-dependent way. The findings of a three-way factorial ANOVA of larval instars performed at varied doses and time intervals, as shown in (S3 Table), revealed substantial variations in larval mortality (P< 0.05).

The current research found that the LC_50_ of *M*. *piperita* ethanol extract was significantly lower than that of other plant extracts, indicating that *M*. *piperita* is more hazardous than other experimental plants. *Cassia fistula* methanol-ethanol extracts (ratio) had LC_50_ of 19.97 and 22.57 mg/L against *A*. *stephensi* and *C*. *quinquefasciatus*, respectively, in a study [59]. In the fourth larval stage of *C*. *quinquefasciatus*, ethanolic and petroleum ether extracts of *Caesalpinia bonduc* caused 100% mortality. In another investigation, chloroform and methanolic extracts of *S*. *villosum* leaves had LC_50_ values of 24.20 to 33.73 ppm after 24 hrs and 23.47 to 30.63 ppm after 48 hrs of continuous exposure against all *A*. *subpictus* larval instars [60, 61].

Microwave-assisted extraction is more efficient for this purpose than traditional methods since it allows for the improvement of numerous essential oil properties, such as toxicity, antibacterial activity, and larvicidal activity. Other Lamiaceae species have been discovered to have the major compounds observed, as well as larvicidal activity against mosquito species, showing that *Cx*. *quinquefasciatus* possesses a defensive and attack strategy [62]. [63] found comparable results for *M*. *piperita* L. essential oil’s larvicidal efficacy against *Cx*. *quinquefasciatus*, where several of the key components found in this study were present. Carvacrol, p-cymene, linalool, -terpinene, and thymol, among other monoterpenes from *C*. *hortense* (L.) Kuntze and Thymus sp., have showed action against *Cx*. *pipiens*.

Biochemical analysis of experimental larvae revealed that the extract treated larvae had much lower carbohydrates, proteins, and lipid profiles than the control group. This shows that plant extracts at 400 ppm concentrations include some harmful elements that impacted the biochemistry of larvae and resulted in substantial mortality. It’s possible that has the ability to changes energy absorption, peroxidation, and insecticidal concern [64]. These findings are consistent with Sharma *et al*. [65], who stated a reduction in *Tenebrio molitor* oocyte hemolymph and fat components after exposure to Malathion.

Microscopic examinations of treated larvae revealed that plant extracts caused sluggish spinning, loss of stability, and gut rupturing, all of which were fatal. As compared to control, this could be attributable to a decline in glucose levels. It’s also possible that the reduction in scavenging presentation, restricted depletion of feeding material, and deprivation of body wall gut is related to insecticidal pressure induced by these extracts.

Brine shrimps lethality assay is a primary study for the validation of anticancer activity [66]. Cancer is one of the thought-provoking ailments of the existing age for investigators. For the treatment of cancer from natural sources, various inventors are demanding the detection and improvement of effective and harmless medications [67]. The brine shrimp cytotoxic bioactivity showed that the plant extract owns a strong toxicological influence (10 ppm LC_50_) and more studies may indicate the separation of active compounds responsible for cytotoxic. In the DPPH, TAC, and FRP activity, the ethanolic leaves extracts of *M*. *piper*ita leaves demonstrated strong antioxidant activity.

Plants are the central basis of potentially beneficial bioactive components for the improvement of new chemotherapeutic agents [68]. In the whole world, Scientists are learning about the benefits of using pharmacologically active chemicals found in medicinal plants [69]. Herbal medicines are used by 80% of people worldwide, due to the great effectiveness, low cost, and fewer side effects of therapeutic plants, [70].

As a result, menthol is the investigation’s principal major component, which is consistent with several other studies [71]. Due to variances in species and chemotypes, plant age, maturity season, extraction process, and geographical origin, the composition of *M*.*piperita* varies greatly. The antibacterial activities of *M*. *piperita* are well-known. These antibacterial properties are mostly due to its chemical composition, which is abundant in oxygenated monoterpenes (92.95%), which have a better effectiveness and a wider spectrum of antimicrobial activity [72].1,2-benzenedicarboxylic acid is used to make polyesters with aliphatic diols as comonomers. The polymer is a crystalline, high-melting material that forms extremely strong fibers [73].

The phytochemical analysis of *M*. *piperita* leaves revealed that it contains a diverse range of bioactive and useful elements, including phenols, alkaloids, flavonoids, reducing sugar, cardiac glycosides, carbohydrates, and steroids [74, 75].

By GC-MS and HP-LC, the active constituents can be isolated from the leaves extracts of the *M*. *piperita*,. The current innovative study recommends that ethanolic and water extracts of *M*. *piperita* are a strong, valuable agent. Larvicidal efficacy along with other properties as an analgesic, anti-inflammatory activity, antibacterial, and antimicrobial, anticancer [76].

So in the existing study, the characterization of *M*. *piperita* was analyzed by using UV-Vis Spectroscopy, FT-IR spectroscopy, and GC-MS analysis. The UV-Vis spectrum profile was selected at the wavelength of 200 to 800 nm and showed the maximum absorption at (209.509) 2.338, and (2825.814) 0.796 respectively. Similar peaks spectrum were reported by [77] in leaf extract of *Meizotropis pellita*.

In my study, FT-IR studied the occurrence of functional groups e:g carboxylic acids, amides, polysaccharides, amines, organic hydrocarbons, and sulfur derivatives halogens. Related active components responsible for multiple therapeutic characteristics of *Aerva lanata* that also worked in methanol leaf extracts of *Ichnocarpus frutescens* were discovered in the infrared region based on peak values [78].

For the comparison of their mass spectrum according to the standard library of Nist, the GC-MS analysis of *M*. *piperita* leaves extracts characterized ten phytocomponents. Of the five compounds identified, the most dominant compounds were t-Butyl hydrogen phthalate (13.99%), 2H-Pyran-2,4(3H)-dione,3-ethyl-5,5-dimethyl -6-phenyl (13.15%), 1,2-Benzenedicarboxylic acid, mono-(2-Ethylhexyl) ester (12.45%), Olean-12-en-28-oic acid, 2.beta., 3.beta.,23-trihydroxy-methyl ester (10.74%), 1,2-Benzene dicarboxylic acid, butyl octyl ester (10.36%), 8-Octadecen-1-ol acetate (9.64%). Among the compounds, three compounds were reported to have antimicrobial activity. In addition to antimicrobial activity, Pentadecanal, 8-Octadecen-1-ol acetate, 2-Cyclohexen-1-one,5-methyl-2-(1-methylethyl), %), Olean-12-en-28-oic acid, 2.beta., 3.beta.,23-trihydroxy-methyl ester was also reported to have antioxidant and anti-cancer properties.

The enzyme NS3 protease which plays an important role in larvicidal efficacy was designated as a target for docking study. A total of ten compounds were used as ligands in docking and evaluated based on binding affinities to justify their larvicidal potential. In current docking, different forces are present between the best-docked compounds i.e., 1, 2-Benzenedicarboxylic acid and 3-ethyl-5, 5-dimethyl -6-phenyl, and the target protein. The two hydrogen bonds are present between each compound and target protein. Hydrogen bonds are very important in docking as they act as the facilitator of protein-ligand binding and are important in protein folding [79]. Besides these, some other forces like Van Der Waals forces, alkyl and Pi-alkyl interactions are present in the ligand-protein interacted complex. Vander wall forces stabilize the formed complex by the creation of a strong cohesive environment. The alkyl and Pi-alkyl interactions are crucial for charge transfer and help in inserting the drug into the binding site of the receptor. The use of molecular docking has aided in the discovery of innovative tiny drug-like scaffolds with the best binding selectivity and affinity for the target [80].

## Conclusion

The current research indicated that for the control of mosquito larvae, *M*. *piperita* could be an active substitute for man-made insecticides. Also, in resident zones of Pakistan, these floras are easily accessible and inexpensive. The *M*. *piperita* reveals strong larvicidal, insecticidal, cytotoxic, and adequate antioxidant capabilities. This study leads that further explorations are necessary for the separation, identification, and characterization of bioactive compounds from *M*. *piperita* exhibiting larvicidal potential. The ethanolic extract of *M*. *piperita* may be directly used at the breeding sites of mosquitoes in stagnant water and localized conditions. It has antilarval potential and can provide a renewable source of natural bioactive compounds. In the future, insecticides of plant sources may suggest appropriate substitutes for synthetic approaches. Among all identified compounds, 1, 2-Benzenedicarboxylic acid, and 3-ethyl-5, 5-dimethyl -6-phenyl are ideal for the production of the novel drug against larvicidal potential due to their improved interaction with the target during docking.

## Supporting information

S1 File (DOCX)

S1 Fig GC-MS chromatograph of ethanolic leaf extract of *M*. *piperita*. (DOCX)

S1 Table Summary of different qualitative phytochemical tests conducted for the analysis of *M*. *piperita*. (DOCX)

S2 Table Preliminary different quantitative phytochemical tests showed for the study of *M*. *piperita*. (DOCX)

S3 Table Three way ANOVA of different hours of exposure, plant and concentrations as variables. (DOCX)

S4 Table UV-Vis peak values of ethanol extract of *M*. *piperita*. (DOCX)

S5 Table Phytochemicals identified in ethanolic leaves extract of *M*. *piperita* by GC-MS peak report. (DOCX)
