# Supplementary material for: Biocidal action, characterization, and molecular docking of Mentha piperita (Lamiaceae) leaves extract against Culex quinquefasciatus (Diptera: Culicidae) larvae
Source: PLoS One. 2022 Jul 14;17(7):e0270219. doi: 10.1371/journal.pone.0270219 (PMC9292459; doi:10.1371/journal.pone.0270219)
Supplement: S1 File — (DOCX) [file pone.0270219.s001.docx]

**LIST OF ABBREVIATIONS**

| Sr. No | NAME | ABBREVIATIONS |
| --- | --- | --- |
| 1 | Bovine serum albumin | BSA |
| 2 | (2,2-diphenyl-1-picrylhydrazyl) | DPPH |
| 3 | Ferric reducing power | FRP |
| 4 | Fourier-transform infrared spectroscopy | FT-IR |
| 5 | Gas chromatography mass spectrometry | GC-MS |
| 6 | Inhibitory concentration | IC |
| 7 | Lethal concentration | LC |
| 8 | Lower confident limit | LCL |
| 9 | Milligram per deciliter | mg/dl |
| 10 | Microgram per milliliter | µg/ml |
| 11 | Molecular operating environment | MOE |
| 12 | One-way analysis of variance | ANOVA |
| 13 | Parts per million | PPM |
| 14 | Standard deviation | STD |
| 15 | Total antioxidant capacity | TAC |
| 16 | Upper confident limit | UCL |
| 17 | Ultraviolet visible spectroscopy | UV-VIS |
