# Supplementary figures and images for: Biocidal action, characterization, and molecular docking of Mentha piperita (Lamiaceae) leaves extract against Culex quinquefasciatus (Diptera: Culicidae) larvae
Source: PLoS One. 2022 Jul 14;17(7):e0270219. doi: 10.1371/journal.pone.0270219 (PMC9292459; doi:10.1371/journal.pone.0270219)

***
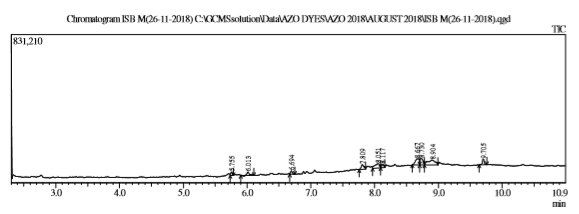
***

**Fig 1. GC-MS chromatograph of ethanolic leaf extract of *M. piperita*.**

Supplement: S1 Fig — (DOCX) [file pone.0270219.s002.docx]
