# Supplementary material for: Biocidal action, characterization, and molecular docking of Mentha piperita (Lamiaceae) leaves extract against Culex quinquefasciatus (Diptera: Culicidae) larvae
Source: PLoS One. 2022 Jul 14;17(7):e0270219. doi: 10.1371/journal.pone.0270219 (PMC9292459; doi:10.1371/journal.pone.0270219)
Supplement: S1 Table — (DOCX) [file pone.0270219.s003.docx]

**S1 Table: Summary of different qualitative phytochemical tests conducted for the analysis of *M. piperita.***

| **Sr. No** | **Secondary Metabolite** | **Name of test** | **Methodology** | **Results** | **References** |
| --- | --- | --- | --- | --- | --- |
|  | Alkaloids | Mayer ‘s test | 2 ml conc. HCl + 2 ml of extract + few drops of Mayer ‘s reagent | White precipitate or green color | **^[31]^** |
|  |  | Hager ‘s test | 1ml of extract + few drops of dilute HCL + 1ml of Hager ‘s reagent | Yellow ppt. | **^[31]^** |
|  |  | Wagner ‘s test | 1 ml of extract + 1 ml of Wagner’s reagent | Reddish brown ppt. | **^[32]^** |
|  | Carbohydrates | Molisch ‘s test | 1 ml extract + 5 ml dist. H_2_O + 1 drop of Molisch ‘s reagent + 1 ml H_2_SO_4_ | Reddish violet ring at the junction | **^[31]^** |
|  |  | Fehling ‘s test | 1 ml of filtrate + 5-8 drops of Fehling‘s solution + heated on a water bath for half an hour. | Brick red precipitation color | **^[33]^** |
|  | Flavonoids | Alkaline reagent test | 2 ml of 2% NaOH + 2 ml of extract + dil. HCl | Yellow solution with NaOH , Turns colorless with dil. HCl | **^[34]^** |
|  |  | FeCl_3_ test | Few drops of FeCl_3_ + 1 ml of extract | Blackish red ppt. | **^[31]^** |
|  | Cardiac glycosides | Liebermann-Burchard’s test | 2 ml of acetic acid + 2 ml of Chloroform + 2 ml of extract + mixture was then cooled + added a few drops of conc. H_2_SO_4_ | Green color | **^[35]^** |
|  |  | Salkowski’ s test | 2 ml of dil. H_2_SO_4_ + 2 ml of extract | Reddish brown color | **^[35]^** |
|  |  | Keller - Kiliani test | 2 ml filtrate + 1 ml glacial acetic acid + 1 ml ferric chloride + 1 ml conc. H_2_SO_4_ | Green blue coloration | **^[36]^** |
|  | Saponins | Frothing test Foam test | 1 ml filtrate + 4 ml dist. H_2_O +mixed well and shaken vigorously | Foam formation | **^[36]^** |
|  |  | Lead acetate test | 1 ml filtrate + 1ml ammonia solution + 1 ml lead acetate + mixed well and shaken vigorously | Black green ppt. or drop green foam | **^[31]^** |
|  | Tannins | FeCl_3_ test | 2 ml of 5% FeCl_3_ + 1 ml extract | Greenish black or dark blue color | **^[35]^** |
|  |  | Alkaline reagent test | 1 ml of 1N NaOH + 1 ml extract | An appearance of yellow to red color | **^[31]^** |
|  |  | Braymer ‘s test | 2 ml of extract + 2 ml H_2_O + 2-3 drops of FeCl_3_ (5%) | Green ppt. | **^[31]^** |
|  | Sterol | Salkowski ‘s test | 5 ml of Chloroform + 2 ml of plant extract + 1 ml of conc.H_2_SO_4_ | Reddish brown color | **^[31]^** |
|  | Quinones |  | 1 ml conc. H_2_SO_4_ + 1 ml extract | Red color | **^[31]^** |
|  | Terpenoids | Salkowski ‘s test | 5 ml extract + 2 ml Chloroform + 3 ml conc. H_2_SO_4_ | Reddish brown color | **^[37]^** |
|  | Phenols | Ellagic acid test | Few drops of 5% glacial acetic acid + 1 ml extract + few drops of 5% NaNO_2_ | Muddy brown color | **^[31]^** |
|  |  |  | 1 ml extract + 1 ml lead acetate | White ppt. | **^[31]^** |
|  | Amino Acid | Ninhydrin test | 1 ml extract + few drops of Ninhydrin reagent | Purple color | **^[31]^** |
|  |  | Xanthoproteic test | 1 ml extract + 1 ml Conc. H_2_SO_4_ | White ppt. | **^[31]^** |
|  | Volatile oil |  | 2 ml extract + 0.1 ml dil. NaOH + dil. HCl + shake the solution | Formation of white ppt. | **^[38]^** |
|  | Starch |  | 1 ml extract + few drops of iodine solution | Color change | **^[31]^** |
|  | Cellulose |  | 1 ml extract + few drops of iodine solution + few drops of conc. H_2_SO_4_ | Brown red color | **^[31]^** |
|  | Anthocyanin and Betacyanin | NaOH test | 1 ml of 2N NaOH + 2 ml of extract + heated at 100 C for about 5 mins. | Bluish green color  **(Anthocyanin)**  Yellow color  **(Betacyanin)** | **^[31]^** |
|  | Anthraquinones | Borntrager ‘s test | 3 ml extract + 3 ml Benzene + 5 ml NH_3_ (10 %) | Pink, Violet or red coloration in Ammonical Layer | **^[34]^** |
|  | Coumarins |  | 1 ml of 10 % NaOH + 1 ml of extract | Yellow color | **^[35]^** |
|  | Phlobatannins | Precipitate test | 1 ml extract + 1 ml HCl (10%) + heat | Red ppt. | **^[37]^** |
|  | Steroid and Phytosteroid |  | 1 ml extract + equal volume of Chloroform + few drops of conc. H_2_SO_4_ | Brown color **(Steroids)**  Bluish –brown ring **(Phytosteroid)** | **^[37]^** |
|  | Leucoanthocyanins |  | 5 ml extract + 5 ml Isoamyl alcohol | Organic layer into a red | **^[31]^** |
