# Supplementary material for: Biocidal action, characterization, and molecular docking of Mentha piperita (Lamiaceae) leaves extract against Culex quinquefasciatus (Diptera: Culicidae) larvae
Source: PLoS One. 2022 Jul 14;17(7):e0270219. doi: 10.1371/journal.pone.0270219 (PMC9292459; doi:10.1371/journal.pone.0270219)
Supplement: S2 Table — (DOCX) [file pone.0270219.s004.docx]

**S2 Table: Preliminary different quantitative phytochemical tests showed for the study of *M. piperita.***

| Sr. No | Secondary metabolite | Methodology | Absorbance | Reference |
| --- | --- | --- | --- | --- |
| 1 | Alkaloids | 1 ml filtrate + 5 ml of 60% H_2_SO_4_ + after 5 mins mix 5 ml of 0.5 % formaldehyde + allowed to stand for 3 hrs. | Absorbance was read at 565 nm. | [39] |
| 2 | Tannins | 0.5 ml of sample extract + 3.7 ml of distilled water + 0.25 ml of Folin Phenol reagent + 0.5 ml of 35% sodium carbonate solution. | Absorbance was measured at 725 nm. | [40] |
| 3 | Flavonoids | 5 m l filtrate + 5 ml dilute ammonia + shaken for 5 mins and upper layer was collected. | Absorbance read at 490 nm. | [39] |
| 4 | Terpenoids | 2.5 ml filtrate + 2.5 ml of 5 % aqueous phosphomolybdic acid solution +2.5 ml of concentrated H_2_SO_4_+ mixed. The mixture was left to stand for 30 mins and then made up to 12.5 ml with ethanol. | Absorbance was taken at 700 nm. | [39] |
| 5 | Proteins | 100 μl of the sample extract + 3 ml of Bradford’s reagent + incubate in the dark for 5mins. | Absorbance was measured at 595 nm | [40] |
| 6 | Steroids | 2 ml filtrate + 2 ml of chromagen solution + solution left to stand for 30 mins. | Absorbance was read at 550 nm. | [39] |
| 7 | Cardiac Glycosides | 1 ml filtrate + 4 ml of alkaline pirate solution + mixture was boiled for 5 mins and allowed to cool. | Absorbance was read at 490 nm. | [39] |
| 8 | Reducing sugar | 1 ml filtrate + 1 ml alkaline copper reagent + mixture was boiled for 5 mins and allowed to cool + 1 ml of phosphomolybdic acid reagent + 2 ml of distilled water. | Absorbance read at 420 nm. | [39] |
| 9 | Carbohydrates | 1 ml of sample solution + 1 ml of 5% phenol + 5 ml of concentrated Sulphuric acid + mix well + leave for 10 mins. | Measure the absorbance at 488 nm. | [40] |
| 10 | Saponins | 10 g dried fine particles, plant sample + 50 ml of 20 % aqueous ethanol + heated (55˚C) on a water bath for 4 hrs with continuous stirring + filtered and the residue re-extracted with another 100 ml of 20 % ethanol + extract was further reduced to 20 ml over a hot water bath (90˚C) + conc. extract was transferred into a 250 ml separating funnel +10 ml diethyl ether + shaken vigorously. Ether layer was discarded and the aqueous layer was collected + purification was repeated +30 ml n-butanol + combined n-butanol extract was washed twice + 5 ml 5 % aqueous sodium chloride +remaining solution was heated in a water bath + After evaporation, the samples were dried in the oven and weighed. |  | [41] |
| 11 | Phenols | 5 ml filtrate+ 0.5 ml Folin calculates reagent + allowed to stand for 30 mins + 2 ml of 20 % sodium carbonate was added. | Absorbance measured at 650 nm. | [39] |
