# Supplementary material for: Biocidal action, characterization, and molecular docking of Mentha piperita (Lamiaceae) leaves extract against Culex quinquefasciatus (Diptera: Culicidae) larvae
Source: PLoS One. 2022 Jul 14;17(7):e0270219. doi: 10.1371/journal.pone.0270219 (PMC9292459; doi:10.1371/journal.pone.0270219)
Supplement: S3 Table — (DOCX) [file pone.0270219.s005.docx]

**S3 Table: Three way ANOVA of different hours of exposure, plant and concentrations as a variables.**

| Source of variation | Sum of squares | | DF | | Mean squares | | F-value | |
| --- | --- | --- | --- | --- | --- | --- | --- | --- |
|  | Ethanol | water | Ethanol | Water | Ethanol | Water | Ethanol | Water |
| Concentrations | 6083 | 5819 | 5 | 5 | 1216.6 | 1163.9 | 445.11 | 457.68 |
| Hours | 1760 | 1783 | 3 | 3 | 586.7 | 594.2 | 214.66 | 233.67 |
| Instars | 259 | 211 | 3 | 3 | 86.3 | 70.3 | 31.56 | 27.63 |
| Residuals | 754 | 702 | 276 | 276 | 2.7 | 2.5 | - | - |

**DF= degree of freedom**
