# Supplementary material for: Biocidal action, characterization, and molecular docking of Mentha piperita (Lamiaceae) leaves extract against Culex quinquefasciatus (Diptera: Culicidae) larvae
Source: PLoS One. 2022 Jul 14;17(7):e0270219. doi: 10.1371/journal.pone.0270219 (PMC9292459; doi:10.1371/journal.pone.0270219)
Supplement: S4 Table — (DOCX) [file pone.0270219.s006.docx]

**S4 Table: UV-Vis peak values of ethanol extract of *M. piperita*.**

| Sr. No | Wavelength (nm) | Absorbance |
| --- | --- | --- |
| 1 | 209.509 | 2.338 |
| 2 | 282.814 | 0.796 |
