# Supplementary material for: Biocidal action, characterization, and molecular docking of Mentha piperita (Lamiaceae) leaves extract against Culex quinquefasciatus (Diptera: Culicidae) larvae
Source: PLoS One. 2022 Jul 14;17(7):e0270219. doi: 10.1371/journal.pone.0270219 (PMC9292459; doi:10.1371/journal.pone.0270219)
Supplement: S5 Table — (DOCX) [file pone.0270219.s007.docx]

**S5 Table: Phytochemicals identified in ethanolic leaves extract of *M. piperita* by GC-MS peak report.**

| **Peak No** | **Retention time** | **Name of the**  **compound**  **IUPAC/Common name** | **Molecular formula** | **Molecular weight** | **Peak area %** | **Compound nature and uses** | **Biological activity** | **Structure** |
| --- | --- | --- | --- | --- | --- | --- | --- | --- |
| **1** | 5.755 | 2-Butanone, 4-(2,6,6-trimethyl-1-cyclohexen-1-yl)- | C_13_H24O | 194 | 7.75 | Terpenes,  Flavouring Agent. | Antimicrobial, anticancer. | 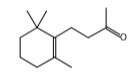 |
| **2** | 6.013 | 2-Napthalenamine ,1,2,4a,5,6,7,8,8a-octahydro-4a-methyl | C_11_H_19_N | 165 | 7.29 | Insecticidal and pest  Repellent. | Antibacterial, Anti-inflammatory ,  Analgestic, antimicrobial. | 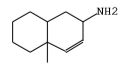 |
| **3** | 6.694 | 2-Cyclohexen-1-one,5-methyl-2-(1-methylethyl) | C_10_H_16_O | 152 | 7.45 | Monoterpene ketone, production of [synthetic](https://en.wikipedia.org/wiki/Chemical_synthesis) [menthol](https://en.wikipedia.org/wiki/Menthol) . | Antimicrobial, antioxidant | 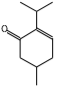 |
| **4** | 7.809 | 1,2-Benzene dicarboxylicacid, butyl octyl ester | C_20_H_30_O_4_ | 334 | 10.36 | Plasticizer compound, lubricant. | Antibacterial | 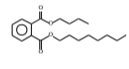 |
| **5** | 8.051 | 8-Octadecen-1-ol acetate | C_20_H_38_O_2_ | 310 | 9.64 | Oleic alcohol, cosmetic agent. | Antioxidant | 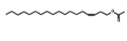 |
| **6** | 8.117 | Pentadecanal | C_15_H_30_O | 226 | 7.48 | 2,3-saturated fatty  aldehyde, fragrance use. | Antioxidant | 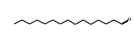 |
| **7** | 8.667 | 2H-Pyran-2,4(3H)-dione,3-ethyl-5,5-dimethyl -6-phenyl | C_18_H18O_3_ | 246 | 13.15 | Heterocyclic, nonaromatic compound, treatment of dental infections. | Antifungal, phytotoxic | 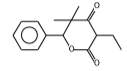 |
| **8** | 8.730 | t-Butyl hydrogen  phthalate | C_12_H_14_O_4_ | 222 | 13.99 | Ester, Plasticizer. | Antioxidant | 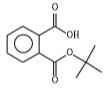 |
| **9** | 8.904 | Olean-12-en-28-oic acid, 2.beta.,  3.beta.,23-trihydroxy-methyl ester | C_31_H_50_O_5_ | 502 | 10.74 | Ester compound,   emulsifying or  solubilizing agent. | Anticancer, antioxidant. | 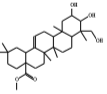 |
| **10** | 9.705 | 1,2-Benzenedicarboxylic acid, mono-(2-  ethylhexyl) ester | C_16_H_22_O_4_ | 278 | 12.45 | Plasticizer compound/  Phthalate ester,  stabilizers and  lubricants. | Antimicrobial,  Mutagenic. | 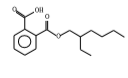 |
